# Duration of same-species antimicrobial non-susceptibility persistence in urinary gram-negative bacilli: a 12-year within-patient cohort study

**DOI:** 10.3389/fcimb.2026.1924619

**Published:** 2026-09-11

**Authors:** Mohammed Alshehri, Ibrahim Tawhari, Mona M. Alshahrani, Ali M. Somily, Khalifa Binkhamis, Ghassan S. Alaskar, Esmahan Tahtouh, Nawar Said Tayfour, Ayman Nasr Tawifik Khalil, Yahya Shabi

**Affiliations:** 1 MD Nephrology Section, Internal Medicine Department, College of Medicine, King Khalid University, Abha, Saudi Arabia; 2 Department of Medicine, College of Medicine, King Khalid University, Abha, Saudi Arabia; 3 Department of Pathology, College of Medicine, King Saud University and King Saud University Medical City, Riyadh, Saudi Arabia; 4 Department of Pathology, College of Medicine, King Saud University, Riyadh, Saudi Arabia; 5 Department of Internal Medicine, Aseer Central Hospital, Abha, Saudi Arabia; 6 Department of Nephrology, Saudi German Hospital, Khamis Mushait, Saudi Arabia; 7 Department of Microbiology and Clinical Parasitology, College of Medicine, King Khalid University, Abha, Saudi Arabia

**Keywords:** antimicrobial stewardship, cohort studies, bacterial drug resistance, gram-negative bacteria, microbial sensitivity tests, prior urine-culture, Saudi Arabia, urinary tract infections

## Abstract

**Introduction:**

Prior urine-culture results can guide empiric antimicrobial therapy, but their value depends on whether the same organism recurs and the prior phenotype remains informative. We estimated the duration of same-species antimicrobial non-susceptibility persistence among urinary gram-negative bacilli in a tertiary-care cohort.

**Methods:**

We conducted a retrospective longitudinal cohort study of urine cultures from patients aged ≥12 years at Aseer Central Hospital, Saudi Arabia, from 2013 to 2024. Consecutive within-patient, same-species isolate pairs were formed for gram-negative bacilli. Among pairs in which the prior isolate was non-susceptible and the next same-species isolate was class-evaluable, persistence was defined as non-susceptibility of the next isolate to the same drug class. Estimates were stratified by organism, drug class, and time since the prior isolate; uncertainty was addressed using patient-clustered bootstrap or Wilson intervals, and temporal decay used modified Poisson regression.

**Results:**

The source cohort included 14,587 eligible urinary gram-negative bacilli cultures from 8,045 patients. The trajectory cohort included 2,401 patients with ≥2 eligible cultures, yielding 4,332 consecutive same-species pairs from 1,745 patients; 3,380 pairs from 1,368 patients entered class-evaluable persistence analyses. *Escherichia coli* and *Klebsiella pneumoniae* met support thresholds for organism-specific inference. Fluoroquinolone non-susceptibility persistence declined from 96.8% within 30 days to 79.9% after 365 days in *E. coli* and from 93.2% to 77.1% in K. pneumoniae. Extended-spectrum cephalosporin non-susceptibility declined from 90.0% to 48.2% in *E. coli* and from 94.9% to 63.6% in K. pneumoniae. Carbapenem non-susceptibility persistence in *K. pneumoniae* declined from 86.8% to 41.9%. All supported organism-class decay models had risk ratios below 1.0 per doubling of time.

**Discussion:**

Prior same-species urinary gram-negative bacilli non-susceptibility carried substantial empiric memory, especially for fluoroquinolones, but persistence declined with time and differed by organism and drug class. Prior results should be interpreted as time-dependent, species- and class-specific evidence, not as an indefinite resistance flag.

## Introduction

Empiric therapy for suspected urinary tract infection is often selected before the current culture and susceptibility report is available (Dunne et al., 2022). During that interval, clinicians draw on the clinical and microbiological information at hand—such as illness severity, recent antimicrobial exposure, local resistance patterns, and the patient’s own microbiology history—when selecting empiric therapy; urine culture and susceptibility testing remain central to optimizing treatment of urinary tract infection (Alkhawaldeh et al., 2022). Prior urine-culture results are especially useful because they are patient-specific and usually available before the current isolate is identified (MacFadden et al., 2014; Honda et al., 2025). Their value, however, depends on a sequence of events: the patient must be re-cultured, the subsequent culture must grow a relevant organism, and the prior susceptibility phenotype must remain informative for the drug being considered.

Prior work supports the principle that previous urine cultures contain clinically useful information. MacFadden and colleagues showed that prior positive urine cultures predicted subsequent organism identity and susceptibility better than chance, and Honda and colleagues, in a cohort of women with recurrent urinary tract infection in UK primary care, recently showed that antimicrobial resistance at a first urinary tract infection was strongly associated with resistance at a later infection, particularly when the inter-infection interval was short (MacFadden et al., 2014; Honda et al., 2025). These studies justify using previous microbiology as empiric evidence, but they also highlight the need to define the time window over which that evidence remains reliable.

This question is particularly important in Saudi tertiary-care settings, where resistance patterns are heterogeneous and stewardship decisions require local evidence (Alhazmi et al., 2023; Khalid et al., 2025). Saudi studies have reported high rates of extended-spectrum beta-lactamase-producing *Escherichia coli* and *Klebsiella pneumoniae* in tertiary care and shifting resistance among non-fermenting gram-negative bacteria—including rising carbapenem resistance in *Pseudomonas aeruginosa*, with trends that varied by organism—in national data (Somily et al., 2014; 2021). Broader local susceptibility surveillance, including work on nosocomial *Enterococcus* species, reinforces the value of organism-specific longitudinal microbiology rather than reliance on aggregated antibiograms alone (Darraj, 2023; Al Bshabshe et al., 2024). Laboratory workflow can also shape routine microbiology data; a local comparison of blood-culture systems illustrates why platform and process metadata matter when culture-derived evidence is interpreted (Somily et al., 2018), even though that study addressed blood rather than urine cultures.

A practical limitation is that susceptibility persistence and clinical applicability are distinct. A prior non-susceptible isolate may remain a strong warning if the same species recurs, yet it becomes less actionable if the next culture grows another species or if the relevant drug class is not tested. Conversely, a prior result may appear to lose predictive value because of changing organism mix, testing panels, or culture-selection practices rather than true loss of the resistance phenotype. Separating same-species phenotype persistence from next-culture applicability is therefore essential for translating prior microbiology into empiric prescribing or clinical decision support.

We therefore estimated the duration of same-species antimicrobial non-susceptibility persistence among urinary gram-negative bacilli over 12 years at a tertiary-care hospital in Southern Saudi Arabia. We aimed to quantify, by organism, drug class, and time since the prior isolate, the probability that a prior non-susceptible same-species urinary isolate would be followed by non-susceptibility to the same class. We also assessed how often prior non-susceptible results remained applicable to the next urine culture through same-species recurrence and repeat class evaluability.

## Materials and methods

### Study design and setting

This retrospective longitudinal cohort study was designed to estimate the empiric memory window of prior urinary gram-negative bacilli non-susceptibility, defined as the probability that, among patients with a prior same-species urinary isolate non-susceptible to a drug class, the next same-species isolate remained non-susceptible to that class when the next isolate was class-evaluable. The study was performed at Aseer Central Hospital (ACH), a 500-bed tertiary-care center serving the Aseer region of Southern Saudi Arabia. As a culture-based study, it characterized antimicrobial non-susceptibility persistence among positive cultures and did not apply clinical criteria for urinary tract infection to define cases. The Institutional Review Board of the Aseer Health Cluster approved the protocol and waived informed consent because the data were retrospective and de-identified. The study was conducted in accordance with the Declaration of Helsinki (1964) and its later amendments. The study was reported in accordance with the Strengthening the Reporting of Observational Studies in Epidemiology (STROBE) statement (von Elm et al., 2007), the STROBE extension for antimicrobial stewardship (STROBE-AMS) (Tacconelli et al., 2016), and the REporting of studies Conducted using Observational Routinely collected health Data (RECORD) statement (Benchimol et al., 2015).

### Data source, eligibility, and pair derivation

Data were drawn from the hospital clinical microbiology antimicrobial susceptibility database, which captured urine-culture organism identification and categorical susceptibility results beginning in 2012. The analytic study period spanned 1 January 2013 to 16 September 2024; the partial first year of capture in 2012 was excluded, and 2024 was retained through the last available culture date. Eligible cultures were consecutive urine cultures from patients aged 12 years or older that grew gram-negative bacilli. External referral cultures were excluded.

Patients with at least two eligible cultures formed the trajectory cohort for within-patient analyses. The primary analysis did not apply 14-day deduplication, because consecutive within-patient culture sequences were required to define analytic same-species isolate pairs.

The analytic unit was the within-species consecutive isolate pair. For each patient and species, each isolate was paired with the next isolate of the same species in that patient, skipping intervening cultures of other species. This pairing targeted same-species persistence rather than recurrence of any urinary gram-negative bacillus. Because no molecular strain typing was performed, these same-species pairs cannot distinguish persistence of the original strain from reinfection with a genetically distinct strain of the same species (strain typing not performed); throughout this report, “same-species recurrence” therefore denotes species-level, not strain-level, identity. Time since the prior same-species isolate was grouped into five intervals (0–30, 31–90, 91–180, 181–365, and more than 365 days).

### Organism classification and susceptibility testing

Organism identification and susceptibility testing were performed for clinical care by thehospital clinical microbiology laboratory using the VITEK 2 automated system (bioMérieux,Marcy-l’Étoile, France). Finalized organism names and categorical susceptibility interpretations were extracted from the laboratory record. Detailed VITEK card or panel types, software versions, raw minimum inhibitory concentrations, disk-zone diameters, and isolate-level quality-control records were not available in the analytic dataset. The VITEK 2 system was used throughout the study period for both identification and susceptibility testing, and the laboratory is accredited and performs routine internal quality control in accordance with CLSI-recommended procedures. However, specific antimicrobial susceptibility testing card versions, instrument software and Advanced Expert System versions, and isolate-level quality-control records were not retrievable from the analytic dataset, and periodic card or software updates over the 12-year period cannot be excluded (**Supplementary Table 5**). Susceptibility results reported for clinical care were interpreted under Clinical andLaboratory Standards Institute breakpoints in effect over 2013–2024 (Clinical and Laboratory Standards Institute, 2013–2024) and collapsed to a binary scale, with non-susceptible defined as an intermediate or resistant categorical result. Categorical susceptible, intermediate, and resistant interpretations were those finalized by the accredited clinical microbiology laboratory at the time of testing, applying the edition of CLSI M100 then in force for each calendar year (**Supplementary Table 5**). Because raw minimum inhibitory concentrations and zone diameters were not retained in the analytic dataset, isolates could not be retrospectively re-interpreted under a single harmonized breakpoint version; contemporaneous categorical results were therefore used throughout.

### Non-susceptibility phenotypes

For each same-species pair, the index phenotype was non-susceptibility of the prior isolate to at least one tested agent within a drug class, and the follow-up phenotype was non-susceptibility of the next isolate to at least one tested agent within that same class, conditional on the next isolate being evaluable for that class. Three non-mutually exclusive class phenotypes were analyzed: fluoroquinolone (ciprofloxacin or levofloxacin), extended-spectrum cephalosporin (cefotaxime, ceftriaxone, ceftazidime, or cefepime; Enterobacterales only, undefined for non-fermenting gram-negative bacilli), and carbapenem (organism-appropriate carbapenems). Difficult-to-treat non-susceptibility was not treated as a separate class. Because not every next isolate was tested for every class, persistence was separated from evaluability. Each class’s persistence denominator was the prior non-susceptible same-species pairs whose next isolate was tested for that class.

### Outcomes

The outcome was persistence of non-susceptibility within the same species and drug class. The denominator comprised same-species isolate pairs in which the prior isolate was non-susceptible and the subsequent isolate was tested for the same drug class. Persistence was defined as the subsequent isolate remaining non-susceptible. This estimate was conditional on reculture, same-species recurrence, and class evaluability. It therefore describes the prognostic value of a prior result among observed same-species recurrences and does not estimate population incidence of non-susceptibility, the probability of obtaining a follow-up culture, or a causal effect. Because urine cultures were obtained for clinical indications rather than scheduled surveillance, time-to-event, Markov, and incidence frameworks were not used.

The applicability analysis assessed how often a prior non-susceptible result could be applied to the next urine culture. Among prior non-susceptible index cultures with any subsequent urine culture, we estimated the proportion in which the next culture grew the same species and the proportion in which the next culture both grew the same species and was tested for the same drug class. Index cultures without a subsequent culture were excluded from these proportions and counted separately.

### Bias and missing data

Because cultures were obtained for clinical indications rather than scheduled surveillance, repeat-culture selection and changing testing practices were expected. To limit this, we used consecutive same-species pairing, defined class evaluability explicitly, and separated persistence from applicability. Missing susceptibility information was not imputed. Pairs lacking follow-up class evaluability were excluded from the relevant persistence estimate but retained for applicability counts.

### Statistical analysis

Patient and cohort characteristics were summarized using medians with interquartile ranges for continuous variables and frequencies with percentages for categorical variables.

The point estimate for persistence was the pair-level pooled proportion within each organism, drug class, and time interval. For supported cells, uncertainty was estimated using a patient-clustered bootstrap with 10,000 replicates, resampling patients as clusters from the fixed analytic pair dataset; 95% bias-corrected and accelerated confidence intervals were reported. Wilson score intervals were used for sparse or boundary cells, defined as an estimated proportion of at least 0.95 or at most 0.05, or fewer than 10 events in either outcome category.

Temporal decay was estimated using modified Poisson regression with a log link (Zou, 2004), modeling persistence as a function of log2-transformed time since the prior same-species isolate, with robust standard errors clustered by patient. Same-day intervals were assigned a value of 1 day before log transformation. Estimates were reported as risk ratios per doubling of time.

Organism-specific main inference was restricted to species with adequate support for time-stratified estimation, defined as at least 30 evaluable prior non-susceptible same-species pairs in at least 3 of the 5 time intervals, including at least 1 interval beyond 180 days. This support criterion was an investigator-defined, *a priori* pragmatic rule rather than the product of a formal power calculation. The threshold of at least 30 evaluable pairs per cell aligns with the common rule-of-thumb for the normal approximation to a binomial proportion and was intended to keep interval-specific proportion estimates reasonably stable and to mitigate small-sample instability; requiring coverage of at least 3 of the 5 intervals, including at least 1 interval beyond 180 days, ensured that temporal decay could be characterized across both short and long intervals rather than being anchored only to short-interval data. Species not meeting this threshold were treated as descriptive support summaries rather than organism-specific main inference. A class-by-species decay row with fewer than 30 persistent non-susceptible follow-up outcomes or fewer than 30 non-persistent follow-up outcomes was reported as not estimable rather than fitted.

Robustness analyses re-estimated the persistence curves within origin-of-index strata (inpatient, outpatient, and emergency or community), under a restriction to same-species pairs separated by at least 14 days, and under patient-balanced estimation with equal weight per patient, with details provided in the **Supplementary Methods**. Regression-based temporal-decay analyses used two-sided tests, with *P* < 0.05 considered statistically significant. Analyses were performed using StataNow/MP 19.5 (StataCorp LLC, College Station, TX, USA) and Python 3.14.5. Full statistical specifications are provided in the **Supplementary Methods**.

## Results

### Cohort and analytic pair structure

**Figure 1:**
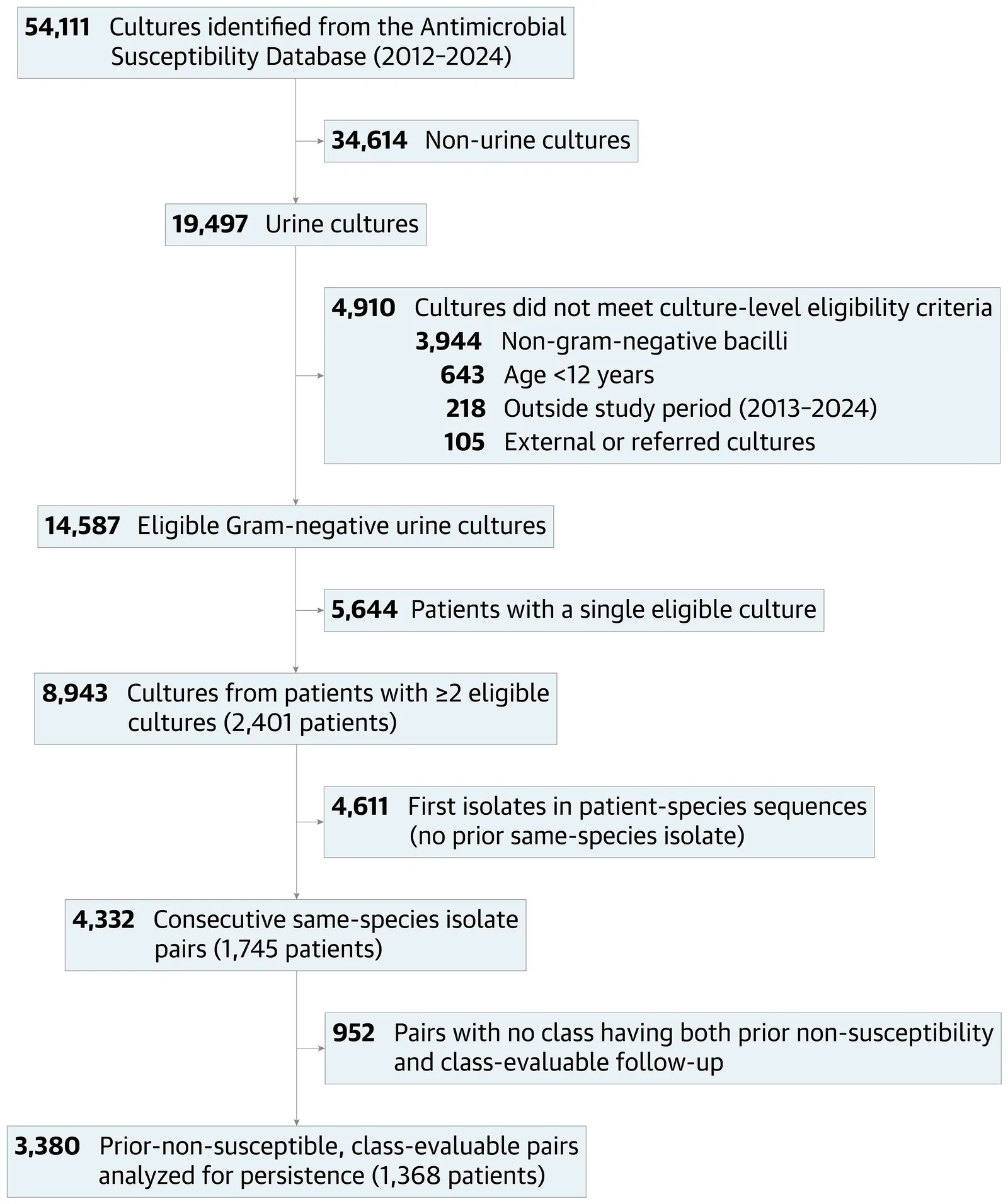
Study flowchart of cohort derivation and same-species pair formation. The flowchart summarizes derivation of the source cohort of eligible urinary gram-negative bacilli cultures, the trajectory cohort of patients with at least two eligible cultures, and the consecutive within-patient, same-species isolate pairs that entered the persistence analyses. “First isolates in patient–species sequences” denotes, for each patient and each species, the first eligible urinary isolate of that species in the patient’s chronological urine-culture sequence—that is, the index isolate that begins each within-patient, same-species trajectory. Each subsequent isolate of the same species is paired with its immediate same-species predecessor, skipping intervening cultures of other species; first isolates are therefore the origin of these pairs and are not themselves counted as follow-up isolates.

The source cohort comprised 14,587 eligible urinary gram-negative bacilli cultures from 8,045 patients from 1 January 2013 to 16 September 2024. Of these, 2,401 patients had at least two eligible cultures and formed the trajectory cohort (8,943 cultures). Consecutive same-species pairing yielded 4,332 pairs from 1,745 patients, of which 3,380 pairs from 1,368 patients had a prior non-susceptible isolate followed by a class-evaluable next isolate and entered the persistence analysis (**Figure 1**). Most pairs occurred at shorter intervals; the median time since the prior same-species isolate was 43 days (IQR, 14–153), with 1,816 pairs separated by 30 days or less and 541 by more than a year.

Trajectory-cohort patients had a median age of 61 years (IQR, 39–75), and 1,148 (47.8%) were women. Of all 2,401 trajectory-cohort patients, the first eligible culture was obtained during inpatient care in 1,368 patients (57.0%), through the emergency department in 862 (35.9%), and in outpatient care in 171 (7.1%). Among the 1,368 inpatients, 1,086 (79.4%) were on general wards and 282 (20.6%) were in the intensive care unit (**Table 1**). *E. coli* was the most common index organism (1,061 patients; 44.2%),followed by *K. pneumoniae* (643; 26.8%), with non-fermenting gram-negative bacilli accounting for a minority of cultures (**Supplementary Table 1**). Within the 4,332-pair analytic set, *E. coli* accounted for 50.4% of pairs and *K. pneumoniae* for 27.5%, with other Enterobacterales contributing 12.1% and non-fermenting gram-negative bacilli 10.0%; *E. coli* and *K. pneumoniae* were the two species meeting the support threshold for organism-specific inference.

**Table 1:** Characteristics of patients contributing to within-patient urinary gram-negative bacilli non-susceptibility persistence analyses.

Characteristic	Patients (*N* = 2,401)
Patient characteristics	
Age, years, median (IQR)	61 (39–75)
Sex, no. (%)	
Men	1,253 (52.2)
Women	1,148 (47.8)
Setting at first culture, no. (%)	
Emergency department	862 (35.9)
Inpatient	1,368 (57.0)
General ward	1,086 (79.4)
Intensive care unit	282 (20.6)
Outpatient	171 (7.1)
Index organism, no. (%)^a^	
* Escherichia coli*	1,061 (44.2)
* Klebsiella pneumonia*	643 (26.8)
* Pseudomonas aeruginosa*	191 (8.0)
* Acinetobacter baumannii*	127 (5.3)
* Proteus mirabilis*	102 (4.2)
* Enterobacter cloacae*	60 (2.5)

Denominators for setting at first culture are nested. Percentages for inpatient (57.0%), emergency department (35.9%), and outpatient (7.1%) care are calculated among all 2,401 trajectory-cohort patients. The general ward (1,086; 79.4%) and intensive care unit (282; 20.6%) percentages are calculated among the 1,368 inpatients only, not among the full cohort. Characteristics are shown at each patient’s first eligible urinary gram-negative bacilli culture among patients with at least two eligible cultures during 2013–2024.

IQR, interquartile range.

^a^ Species shown are the organism at each patient’s first eligible culture; the six mostcommon are listed, and the full species distribution by year is shown in **Supplementary Table 1**.

### Persistence over time

**Figure 2:**
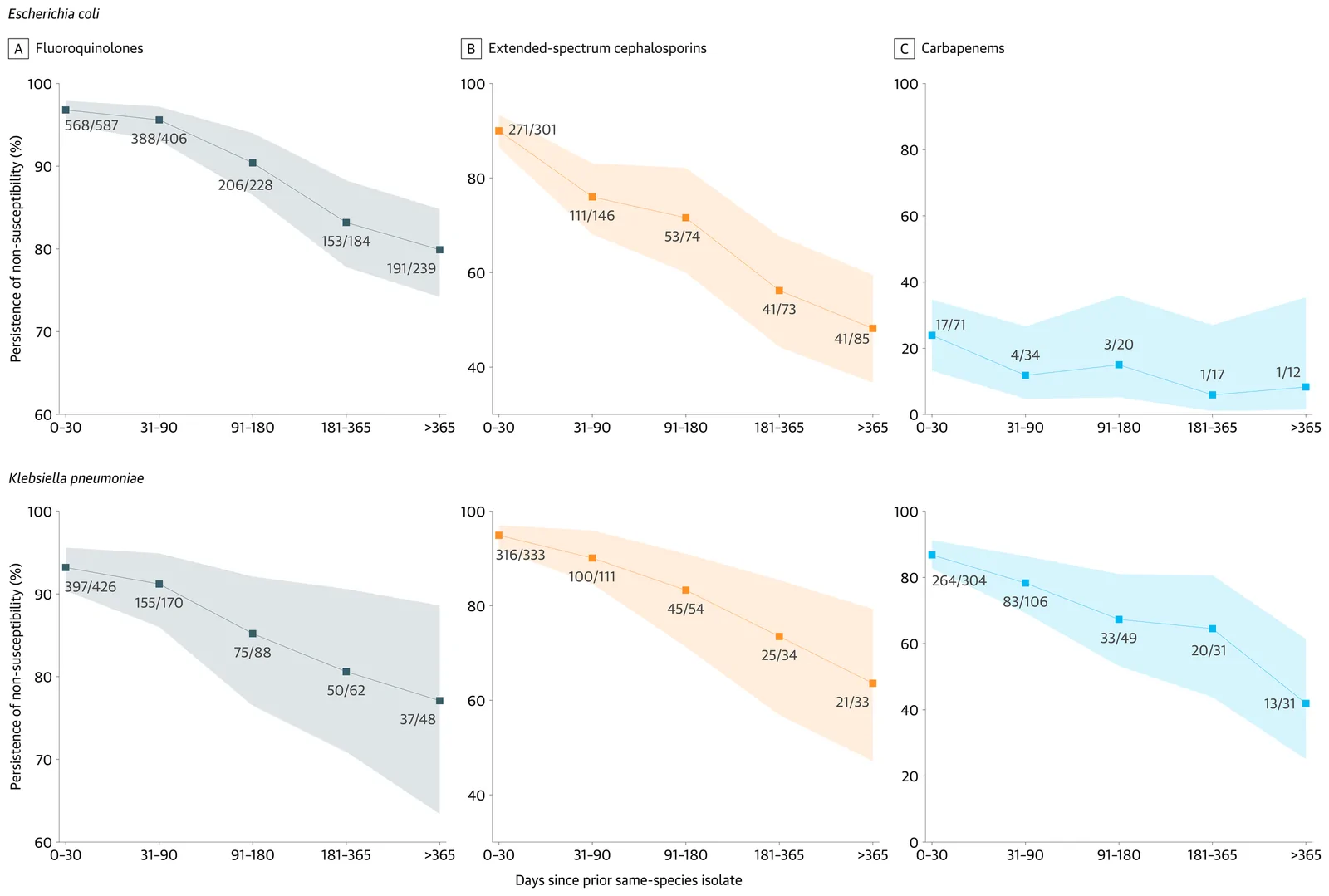
Within-patient persistence of urinary gram-negative bacilli non-susceptibility by drug class, organism, and time since prior same-species isolate. Panels are arranged in columns by antimicrobial class: **(A)** fluoroquinolones, **(B)** extended-spectrum cephalosporins, and **(C)** carbapenems. Within each column, the upper graph shows *Escherichia coli* and the lower graph shows *Klebsiella pneumoniae*. Points are pair-level pooled persistence proportions plotted against days since the prior same-species isolate, with the number of non-susceptible follow-up isolates over the number of class-evaluable pairs shown at each interval; shaded bands are 95% confidence intervals.

Fluoroquinolone non-susceptibility persistence in *E. coli* was 96.8% within 30 days and 79.9% after 365 days, and in *K. pneumoniae*, 93.2% and 77.1%, respectively (**Figure 2**; **Table 2**).

**Table 2:** Within-patient persistence of urinary gram-negative bacilli non-susceptibility, by drug class, organism, and time since the prior isolate.

Drug class and organism^a^	Time since prior, days	Persistence, % (95% CI)^b^,^c^	Prior non-susceptible pairs, no.	Class-evaluable follow-up, no. (%)^d^	Patients, no.
Fluoroquinolone non-susceptibility					
*Escherichia coli*	0–30	96.8% (95.0–97.9)	607	587 (96.7%)	360
	31–90	95.6% (93.1–97.2)	419	406 (96.9%)	279
	91–180	90.4% (85.8–93.7)	229	228 (99.6%)	172
	181–365	83.2% (77.2–88.1)	186	184 (98.9%)	148
	>365	79.9% (74.1–84.9)	242	239 (98.8%)	191
*Klebsiella pneumonia*	0–30	93.2% (90.3–95.4)	438	426 (97.3%)	261
	31–90	91.2% (85.8–94.9)	178	170 (95.5%)	114
	91–180	85.2% (75.9–91.8)	90	88 (97.8%)	63
	181–365	80.6% (69.0–89.7)	64	62 (96.9%)	53
	>365	77.1% (63.8–87.8)	48	48 (100.0%)	44
Extended-spectrum cephalosporin non-susceptibility					
*Escherichia coli*	0–30	90.0% (86.2–93.1)	357	301 (84.3%)	186
	31–90	76.0% (67.8–82.6)	210	146 (69.5%)	113
	91–180	71.6% (59.7–81.3)	110	74 (67.3%)	71
	181–365	56.2% (43.7–67.6)	87	73 (83.9%)	64
	>365	48.2% (36.8–59.8)	129	85 (65.9%)	77
*Klebsiella pneumoniae*	0–30	94.9% (91.9–96.9)	359	333 (92.8%)	203
	31–90	90.1% (83.2–94.8)	141	111 (78.7%)	85
	91–180	83.3% (71.3–91.0)	61	54 (88.5%)	47
	181–365	73.5% (56.9–85.4)	42	34 (81.0%)	33
	>365	63.6% (46.9–80.6)	45	33 (73.3%)	31
Carbapenem non-susceptibility					
*Escherichia coli*	0–30	23.9% (14.9–34.7)	71	71 (100.0%)	64
	31–90	11.8% (4.7–26.6)	34	34 (100.0%)	31
	91–180	15.0% (5.2–36.0)	20	20 (100.0%)	19
	181–365	5.9% (1.0–27.0)	18	17 (94.4%)	17
	>365	8.3% (1.5–35.4)	12	12 (100.0%)	12
*Klebsiella pneumoniae*	0–30	86.8% (82.2–90.6)	306	304 (99.3%)	192
	31–90	78.3% (69.1–85.8)	109	106 (97.2%)	73
	91–180	67.3% (53.1–80.4)	49	49 (100.0%)	40
	181–365	64.5% (48.4–81.2)	31	31 (100.0%)	30
	>365	41.9% (26.7–62.5)	31	31 (100.0%)	30

CI, confidence interval.

^a^ Estimates are restricted to *Escherichia coli* and *Klebsiellapneumoniae*, the only species meeting the support threshold for time-stratifiedorganism-specific estimation. Species-level support is shown in **Supplementary Table 4**. Extended-spectrum cephalosporin non-susceptibility is evaluable in Enterobacterales only.

^b^ Persistence is the percentage of next same-species isolates still non-susceptible to the class, conditional on the next isolate being class-evaluable.

^c^ Confidence intervals for supported cell-specific proportion estimates were calculated using patient-clustered bias-corrected and accelerated bootstrap intervals; sparse or boundary cells used Wilson score intervals.

^d^ Class-evaluable follow-up is the number and percentage of prior non-susceptible same-species pairs whose next isolate was tested for the class; persistence is conditional on this evaluability, separating true decay from declining testing.

Extended-spectrum cephalosporin non-susceptibility persistence in *E. coli* was 90.0% within 30 days and 48.2% after 365 days, and in *K. pneumoniae*, 94.9% and 63.6% respectively (**Figure 2**; **Table 2**). Class-evaluable follow-up for each cell is tabulated in **Table 2**.

Carbapenem non-susceptibility persistence in *K. pneumoniae* was 86.8% within 30 days and 41.9% after 365 days (**Figure 2**; **Table 2**). In *E. coli*, descriptive carbapenem non-susceptibility persistence was 23.9% within 30 days and 8.3% after 365 days.

### Relative decay per doubling

**Figure 3:**
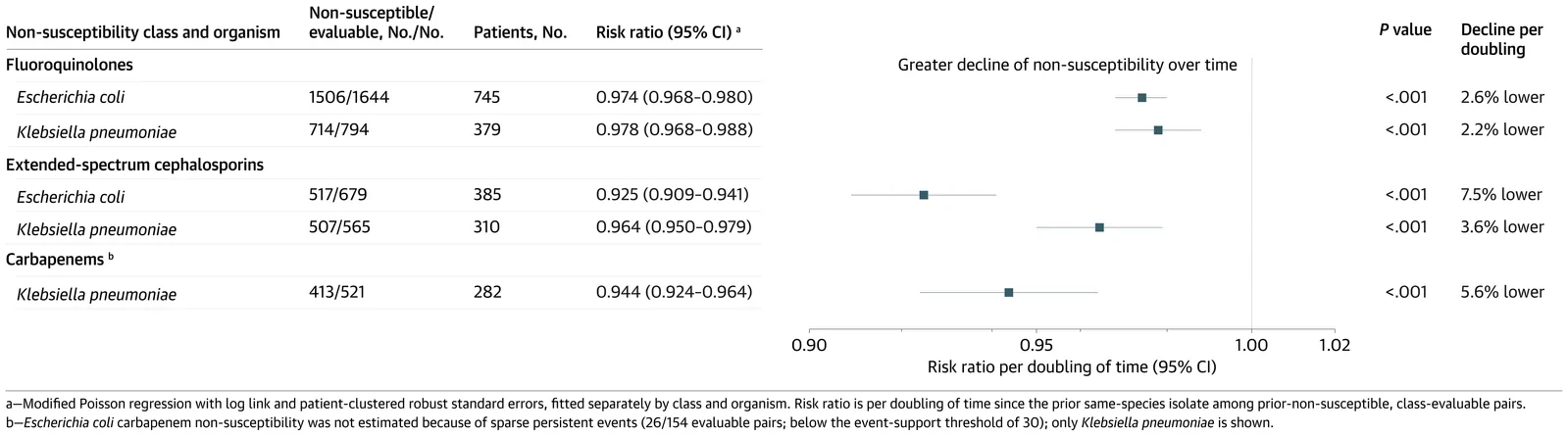
Relative probability of remaining non-susceptible per doubling of time since the prior same-species isolate. Squares indicate risk ratios, and horizontal lines indicate 95% confidence intervals.

For fluoroquinolone non-susceptibility, the risk ratio per doubling of time was 0.974 (95% CI, 0.968–0.980; 2.6% relative reduction) in *E. coli* and 0.978 (95% CI, 0.968–0.988; 2.2% reduction) in *K. pneumoniae* (both *P* < 0.001) (**Figure 3**; **Supplementary Table 2**).

For extended-spectrum cephalosporin non-susceptibility, the risk ratio per doubling was 0.925 (95% CI, 0.909–0.941; 7.5% reduction) in *E. coli* and 0.964 (95% CI, 0.950–0.979; 3.6% reduction) in *K. pneumoniae* (both *P* < 0.001). For carbapenem non-susceptibility, the risk ratio per doubling in *K. pneumoniae* was 0.944 (95% CI, 0.924–0.964; 5.6% reduction; *P* < 0.001); in *E. coli*, it was not estimable, with 26 non-susceptible follow-up outcomes among 154 evaluable pairs (**Supplementary Table 2**).

### Applicability of prior results

For fluoroquinolone non-susceptibility, same-species, class-evaluable recurrence of the next culture was 71.1% for *E. coli* and 71.9% for *K. pneumoniae* within 30 days and 65.0% for *E. coli* and 39.4% for *K. pneumoniae* after 365 days (**Table 3**).

**Table 3:** Applicability of prior non-susceptible urine isolates: same-species recurrence and repeat class evaluability.

Drug class and organism^a^	Measure^b^	Time since prior result, days				
		0–30	31–90	91–180	181–365	>365
Fluoroquinolone non-susceptibility						
*Escherichia coli*	Same-species recurrence, %	73.2	73.4	71.3	70.0	65.8
	Same-species + class-evaluable, %	71.1	71.2	70.9	69.1	65.0
	Index cultures, no. (patients)	776 (468)	504 (342)	275 (210)	207 (169)	260 (220)
*Klebsiella pneumoniae*	Same-species recurrence, %	74.0	59.8	60.2	58.1	39.4
	Same-species + class-evaluable, %	71.9	58.2	59.2	56.8	39.4
	Index cultures, no. (patients)	572 (354)	239 (176)	103 (79)	74 (66)	66 (63)
Extended-spectrum cephalosporin non-susceptibility						
*Escherichia coli*	Same-species recurrence, %	70.3	68.3	69.7	69.5	68.1
	Same-species + class-evaluable, %	58.4	43.7	48.4	57.9	44.2
	Index cultures, no. (patients)	464 (284)	252 (203)	122 (113)	95 (86)	138 (125)
*Klebsiella pneumoniae*	Same-species recurrence, %	72.0	58.1	53.3	60.4	44.3
	Same-species + class-evaluable, %	66.8	44.4	48.0	47.9	29.5
	Index cultures, no. (patients)	479 (297)	198 (155)	75 (69)	48 (44)	61 (60)
Carbapenem non-susceptibility						
*Escherichia coli*	Same-species recurrence, %	67.4	61.4	60.0	68.2	40.0
	Same-species + class-evaluable, %	67.4	61.4	60.0	63.6	40.0
	Index cultures, no. (patients)	95 (85)	44 (41)	25 (23)	22 (22)	15 (15)
*Klebsiella pneumoniae*	Same-species recurrence, %	73.3	61.3	56.6	55.6	51.4
	Same-species + class-evaluable, %	72.8	59.9	56.6	55.6	51.4
	Index cultures, no. (patients)	404 (255)	142 (108)	53 (48)	36 (35)	35 (35)

Same-species recurrence is defined at the species level; strain-level typing was not performed, so recurrent isolates of the same species may represent either persistence of the original strain or reinfection with a genetically distinct strain (strain typing not performed).

^a^ Main inference is limited to *Escherichia coli* and *Klebsiellapneumoniae*, the species meeting the support threshold; species-level support counts forother organisms are shown in **Supplementary Table 4**.

^b^ Index cultures are prior isolates non-susceptible to the stated class that had a subsequent culture of any species. Same-species recurrence is the percentage whose next culture grew the same species; same-species + class-evaluable is the percentage whose next isolate was the same species and was tested for the class. Both percentages use the index-culture denominator. Index cultures without a subsequent culture were excluded from these percentages.

For extended-spectrum cephalosporin non-susceptibility, same-species, class-evaluable recurrence in *E. coli* was 58.4% within 30 days and 44.2% after 365 days, and in *K. pneumoniae*, 66.8% and 29.5%, respectively (**Table 3**). Across non-susceptible index cultures, 22% to 29% had no subsequent culture; these were excluded from the applicability proportions and counted separately.

### Robustness and support

In the inpatient-origin stratum, *E. coli* fluoroquinolone persistence was 97.7%within 30 days and 86.5% after 365 days, and *E. coli* cephalosporin persistence was91.0% and 53.1% (**Supplementary Table 3**). Restricting pairs to those separated by at least 14 days, and patient-balanced estimation,reproduced the primary curves (**Supplementary Table 3**).

Under patient-balanced estimation, *E. coli* fluoroquinolone persistence was 95.7%within 30 days and 76.4% after 365 days, against pair-level pooled values of 96.8% and 79.9% (**Supplementary Table 3**). Only *E. coli* and *K. pneumoniae* met the threshold fororganism-specific estimation; the remaining species are reported as descriptive support counts(**Supplementary Table 4**). The sparsest cells, including long-interval cephalosporin and carbapenem strata and *E. coli* carbapenems, carried the widest confidence intervals (**Table 2**).

## Discussion

In this 12-year within-patient cohort of urinary gram-negative bacilli, prior same-species non-susceptibility retained clear empiric signal, but its strength varied substantially by organism, drug class, and time. Fluoroquinolone non-susceptibility in *E. coli* and *K. pneumoniae* remained highly persistent beyond 1 year. Extended-spectrum cephalosporin non-susceptibility decayed more markedly, particularly in *E. coli*, and carbapenem non-susceptibility in *K. pneumoniae* declined substantially across intervals. The applicability analysis showed why historical results cannot be used automatically: the next culture did not always recover the same species, and repeat class evaluability was incomplete.

These results align with earlier urine-culture prediction studies while adding explicit time-, species-, and drug-class resolution. MacFadden and colleagues found that prior positive urine cultures predicted subsequent organism identity and susceptibility better than chance, and Honda and colleagues, studying women with recurrent urinary tract infection in UK primary care, found that resistance at a first urinary tract infection and shorter inter-infection time were strong determinants of resistance at a later infection (MacFadden et al., 2014; Honda et al., 2025). The present study extends that literature by conditioning persistence on same-species recurrence (strain typing not performed) and class evaluability, thereby separating phenotype persistence from the practical question of whether a prior result can be applied to the next culture.

The distinction between same-species recurrence and same-strain persistence has direct interpretive consequences that our data cannot resolve. The high fluoroquinolone persistence observed beyond 1 year (79.9% in *E. coli* and 77.1% in *K. pneumoniae*) is consistent with a substantial contribution of clonal relapse, in which the original resistant strain persists in the urinary tract or a gastrointestinal reservoir; conversely, part of the temporal decay we observed may reflect sequential reinfection with a genetically distinct strain of the same species. These scenarios carry different clinical implications: relapse with a persisting strain may direct attention to source control, adherence, and the possibility of a persistent reservoir, whereas reinfection argues for re-evaluating empiric selection against the current local flora. Because no strain-level typing (e.g., PFGE, MLST, or whole-genome sequencing) was performed, we cannot quantify the relative contributions of relapse and reinfection, and these interpretations remain hypotheses. Accordingly, all persistence estimates in this study should be read as reflecting same-species, species-level recurrence (strain typing not performed) rather than confirmed microbiological persistence of a single strain.

The findings also fit a Saudi antimicrobial resistance context in which local organism-specific data matter. Studies by Ali Somily and colleagues have shown clinically important resistance burdens among both Enterobacterales and non-fermenting gram-negative bacilli in Saudi settings (Somily et al., 2014, 2021). The present study does not duplicate those surveillance questions; it adds a within-patient temporal layer by asking how long an observed non-susceptible phenotype remains informative for the same species and drug class. Broader Saudi longitudinal susceptibility literature, including local tertiary-care work on nosocomial *Enterococcus* species, supports the same premise: local microbiology should be interpreted by organism and over time, not as a static hospital-wide background rate (Al Bshabshe et al., 2024). Although that work concerned *Enterococcus*, a gram-positive organism outside the scope of our urinary gram-negative bacilli, it is cited only to illustrate the more general principle that organism-specific, longitudinal interpretation is preferable to reliance on aggregated hospital-wide rates.

A biological hypothesis, not tested in this study, may help explain why persistence differed so markedly by drug class. Fluoroquinolone resistance in Enterobacterales is predominantly chromosomally encoded, arising from stepwise target-site mutations in *gyrA* and *parC* with additional contributions from efflux upregulation and, in some isolates, plasmid-mediated quinolone-resistance determinants; such chromosomal changes are vertically inherited and generally stable within a lineage (Redgrave et al., 2014). This is consistent with the high and durable fluoroquinolone persistence we observed. By contrast, extended-spectrum cephalosporin resistance is frequently mediated by mobile *CTX-M*-type extended-spectrum beta-lactamases, and carbapenem resistance by transferable carbapenemase genes, both commonly carried on plasmids that can be gained or lost and that move between lineages (Bevan et al., 2017; Nordmann and Poirel, 2014). Greater mobility and the potential for clonal replacement are consistent with the steeper temporal decay we observed for extended-spectrum cephalosporin and carbapenem non-susceptibility. We emphasize, however, that no molecular data—minimum inhibitory concentrations, resistance gene content, plasmid characterization, or strain typing—were available in this dataset, so these mechanistic explanations remain hypotheses that would require genotypic confirmation.

For antimicrobial stewardship, these results support treating prior urine-culture history as time-dependent evidence rather than as a permanent binary label (Brintz et al., 2024). A recent prior non-susceptible same-species isolate should increase concern for persistence, but the degree of concern should differ by drug class (Mitrani-Gold et al., 2023) and, as the present study shows, by organism. The high persistence observed for fluoroquinolones suggests that prior non-susceptibility may remain relevant for longer intervals, whereas cephalosporin and carbapenem decisions require more caution as time from the prior isolate increases. These data do not define treatment choices by themselves; they should be integrated with illness severity, syndrome, local antibiograms, allergy history, renal function, recent antimicrobial exposure, and current culture results when available. Importantly, microbiological persistence of a non-susceptible phenotype is not itself evidence of ongoing infection, treatment failure, or a mandate to change therapy; it is one time-, species-, and class-specific input that must be weighed against clinical severity, antimicrobial exposure history, individual patient characteristics, and the results of the current culture when available. Because our non-susceptibility definition combined intermediate and resistant results, stewardship applications should also recognize that intermediate isolates may remain treatable with optimized dosing, so a prior non-susceptible flag based on the composite should prompt review of the specific agent and achievable drug exposure rather than automatic exclusion of that class.

The applicability analysis is clinically important because a prior result can fail before it informs treatment: the next culture may grow a different species, or the next isolate may not be tested for the same class. This distinction matters for electronic decision support. A system that simply flags any historical resistance may overwarn clinicians, whereas one that incorporates species, drug class, and time since the prior isolate could produce more calibrated warnings (Yang et al., 2023). Our composite non-susceptibility definition, which combined intermediate and resistant categorical results, may itself contribute to overwarning, because some intermediate isolates can still respond to optimized or high-dose regimens, consistent with the Clinical and Laboratory Standards Institute susceptible-dose-dependent concept, so a warning based on the merged phenotype could overstate the likelihood of clinically meaningful resistance for such agents (Clinical and Laboratory Standards Institute, 2013–2024). Such systems still require local validation because testing panels, laboratory platforms, and culture-ordering behavior affect which phenotypes enter the dataset. A local laboratory-method study focused on blood culture time-to-detection, although outside urinary isolates, reinforces the need to capture platform and workflow details when routinely collected culture data are used for inference (Somily et al., 2018).

This applicability gap widens with time since the prior isolate. Although persistence remained high among evaluable same-species recurrences, the subset of patients to whom a long-interval memory window actually applies shrank as the interval lengthened. Beyond 365 days, the proportion of prior non-susceptible index cultures whose next culture both recovered the same species and remained evaluable for the same drug class fell to roughly 29.5%–65.0% across organisms and classes (for example, 65.0% for fluoroquinolones in *E. coli* and 39.4% for fluoroquinolones and 29.5% for extended-spectrum cephalosporins in *K. pneumoniae*); the 39.4%–51.4% range noted for these long intervals corresponds to particular *K. pneumoniae* cells, whereas the fuller cross-class range extends lower. In addition, 22%–29% of non-susceptible index cultures had no subsequent culture at all and were therefore not eligible for these proportions. Consequently, for a substantial share—often more than half—of prior non-susceptible results at long intervals, the next culture grew a different species or was not tested for the same class. The long-interval memory windows therefore describe high persistence among a diminishing subset of patients rather than durable applicability across the cohort, which tempers any interpretation of uniformly durable high persistence and matters directly for decision support, where a prior-result warning is actionable only when the same species recurs and the relevant class is retested.

This study has several strengths. It used a large routinely collected microbiology dataset spanning more than a decade, defined the analytic unit as consecutive same-species within-patient pairs, separated persistence from class evaluability, and used patient-clustered uncertainty estimates to account for repeated observations. Sensitivity analyses restricting short-interval pairs and balancing patients produced results consistent with the primary estimates.

Several limitations should be considered. The study included positive urine cultures rather than clinically adjudicated urinary tract infections, so the estimate applies to observed culture recurrence rather than infection incidence, clinical syndrome, or treatment response. Because cultures were ordered during clinical care rather than scheduled surveillance, the analysis was subject to selection into testing and repeat testing; clinical indication and syndrome could be incomplete or inaccurate in routinely collected records. The single-center tertiary-care setting in Southern Saudi Arabia may limit generalizability to other hospitals, community settings, or populations with different organism distributions and antimicrobial use. Raw minimum inhibitory concentrations, disk-zone diameters, strain typing, detailed antibiotic exposure, comorbidities, catheter status, clinical syndrome, and platform-level laboratory metadata were not available, and breakpoint or susceptibility-panel changes over time could affect class evaluability. Same-species recurrence does not prove persistence of the same strain, and molecular methods such as PFGE, MLST, or whole-genome sequencing would be required to confirm true microbiological persistence and to distinguish clonal relapse from reinfection with a genetically distinct strain of the same species (Hidad et al., 2022). Finally, the analysis was observational and conditional on reculture, same-species recurrence, and class evaluability; it should not be interpreted as estimating causal effects or population-level rates of resistant urinary infection.

Two features of this design warrant particular caution. First, because the cohort comprised positive urine cultures rather than clinically adjudicated infections, an unknown proportion of isolates likely represented asymptomatic bacteriuria, which is highly prevalent in older adults and can persist as stable colonization for prolonged periods in the absence of therapeutic selective pressure (Nicolle et al., 2019; Biggel et al., 2019). Given the older age of this cohort (median 61 years), such colonizing strains may be carried across successive cultures and inflate apparent same-species non-susceptibility persistence, independently of infection dynamics or treatment response. Our estimates therefore describe the persistence of non-susceptibility across recurrent positive cultures and should not be read as the incidence, or persistence, of symptomatic urinary tract infection; extrapolation to acute symptomatic UTI populations, in whom the balance of true infection versus colonization differs, requires caution. Second, because cultures were obtained on clinical indication rather than by protocolized follow-up sampling, patients who were recultured may represent a subgroup with more complex, recurrent, or persistent infection or colonization; persistence estimated within this recultured population may therefore exceed what would be observed in an unselected population sampled at scheduled intervals.

Several features of the setting make direct transportability uncertain. The cohort was drawn from a single 500-bed tertiary referral center in the Aseer region of Southern Saudi Arabia, with a case mix weighted toward inpatients and intensive-care and other complex patients (57.0% of trajectory-cohort patients had their index culture during an inpatient stay, of whom 20.6% were in intensive care), rather than the predominantly community or primary-care populations in which many empiric urinary tract infection decisions are made. Local organism distribution, antimicrobial use and stewardship patterns, and extended-spectrum beta-lactamase and carbapenemase epidemiology, together with the laboratory’s testing panels, platform, and applied breakpoints, may differ from those in community and primary-care practice, in pediatric populations, in other Saudi regions (Alhazmi et al., 2023; Khalid et al., 2025), and in other countries. Consequently, the absolute persistence percentages and the specific time thresholds (the empiric memory windows) reported here may not transfer directly to other settings, even though the qualitative pattern of declining persistence with increasing time since the prior isolate, together with differences by organism and drug class, is likely to be more broadly generalizable. The specific interval thresholds should therefore be validated locally, ideally with linked clinical and antimicrobial exposure data, before they are embedded in decision support tools or stewardship rules elsewhere.

In particular, we did not have complete longitudinal data on intervening antimicrobial exposure between the index and follow-up isolates, nor on urinary catheterization, recurrent urinary tract infection, comorbidity burden, broader healthcare exposure, or hospitalization history. Because prior antimicrobial exposure is a recognized driver of resistance acquisition and maintenance (Mitrani-Gold et al., 2023), residual confounding cannot be excluded, and the observed temporal decay may partly reflect changes in selective pressure—such as withdrawal of an implicated antibiotic—rather than purely intrinsic biological waning of the resistance phenotype. We considered a formal exposure-stratified subgroup analysis (for example, comparing pairs with and without intervening antimicrobial exposure), but this was not feasible: antibiotic use outside the institution and through non-integrated healthcare encounters could not be reliably captured, so any exposed-versus-unexposed classification would be subject to substantial exposure misclassification. These estimates should therefore be read as describing observed phenotype persistence under routine care rather than the isolated biological durability of resistance.

Because contemporaneous categorical interpretations were used, breakpoint revisions across successive CLSI M100 editions (for example, the 2019 lowering of Enterobacterales fluoroquinolone minimum inhibitory concentration breakpoints in the 29th edition, and earlier revisions to cephalosporin and carbapenem breakpoints around 2010) could have shifted the classification of borderline isolates, so non-susceptibility may not have been defined uniformly across the study period; this is most plausibly a source of non-differential misclassification that would tend to modestly attenuate or shift, rather than spuriously create, the observed persistence estimates. Similarly, although the VITEK 2 platform and routine quality-control practices were used throughout, undocumented card or software updates could have affected a small number of borderline categorical calls, an effect generally expected to be limited for the categorical results used here.

In addition, non-susceptibility was analyzed as a composite of intermediate and resistant categorical results; because the intermediate and resistant categories were irreversibly merged into a single non-susceptible phenotype during construction of the analytic dataset, we could not perform a *post hoc* sensitivity analysis separating the two, and this composite may overwarn for agents where intermediate isolates remain treatable with optimized regimens. Future datasets should retain the full three-level susceptible/intermediate/resistant classification so that the contribution of intermediate results to persistence estimates and to decision-support warnings can be examined directly.

Future work should link microbiology trajectories with antimicrobial exposure, catheterization, comorbidity, hospitalization, source of acquisition, and clinical outcomes. Molecular typing or whole-genome sequencing would help distinguish relapse with the same strain from replacement by another strain of the same species. Multicenter and multinational validation, across Saudi hospitals and other countries, would show whether the memory windows observed here are stable across laboratories, patient populations, and antimicrobial use patterns, and would clarify how far the specific interval thresholds can be generalized beyond this single-center cohort.

## Conclusion

Prior urinary gram-negative bacilli non-susceptibility has measurable, time-dependent empiric value when the next culture grows the same species and is evaluable for the same drug class. Persistence is strongest and most durable for fluoroquinolone non-susceptibility, whereas extended-spectrum cephalosporin and carbapenem non-susceptibility show greater decay in supported organism-class analyses. Clinical decision support and stewardship workflows should treat prior urine-culture results as species-, class-, and interval-specific evidence, with validation in other settings using linked clinical and antimicrobial exposure data.

## Data Availability

The raw data supporting the conclusions of this article will be made available by the authors, without undue reservation.

## Supplementary material

Supplementary Table 1 Distribution of urinary gram-negative bacilli species in the source and trajectory cohorts.

Supplementary Table 2 Modified Poisson regression estimates of non-susceptibility decay per doubling of time, by organism and drug class.

Supplementary Table 3 Sensitivity analysis restricting short-interval pairs.

Supplementary Table 4 Sensitivity analysis balancing patient contribution.

Supplementary Table 5 CLSI M100 breakpoint editions applied by calendar year, 2013-2024.

Supplementary Methods Detailed analytic methods, including pair construction, class-evaluability rules and sensitivity analyses.
