# Supplementary material for: Duration of same-species antimicrobial non-susceptibility persistence in urinary gram-negative bacilli: a 12-year within-patient cohort study
Source: Front Cell Infect Microbiol. 2026 Sep 11;16:1924619. doi: 10.3389/fcimb.2026.1924619 (PMC13612208; doi:10.3389/fcimb.2026.1924619)
Supplement: Supplementary file 1 [file Table1.docx]

**Supplementary Material**

| **Table S1.** Species frequency by year among trajectory-cohort urinary gram-negative bacilli cultures (N = 8,943 cultures)ᵃ | | | | | | | | | | | | | | |
| --- | --- | --- | --- | --- | --- | --- | --- | --- | --- | --- | --- | --- | --- | --- |
| Species | 2013 | 2014 | 2015 | 2016 | 2017 | 2018 | 2019 | 2020 | 2021 | 2022 | 2023 | 2024 ᵇ | Total | % |
| Enterobacterales | | | | | | | | | | | | | | |
| *Citrobacter* *amalonaticus* | — | 3 | 1 | — | — | — | — | — | — | — | — | 1 | 5 | 0.1 |
| *Citrobacter* *braakii* | 2 | — | 1 | — | — | — | — | — | 2 | 3 | — | — | 8 | 0.1 |
| *Citrobacter* *farmeri* | — | 1 | — | — | — | — | 1 | — | — | — | — | 1 | 3 | 0.0 |
| *Citrobacter* *freundii* | 2 | 14 | 6 | 5 | 5 | 3 | — | 3 | 2 | 2 | 6 | 8 | 56 | 0.6 |
| *Citrobacter* *koseri* | 2 | 2 | 2 | 2 | 2 | — | 1 | — | 3 | 1 | 3 | 1 | 19 | 0.2 |
| *Citrobacter* sp. | 1 | 2 | 1 | 1 | 1 | 8 | 7 | 4 | 9 | — | 1 | 5 | 40 | 0.4 |
| *Citrobacter* *youngae* | — | — | — | — | — | — | — | — | 1 | — | 2 | 1 | 4 | 0.0 |
| *Cronobacter* *sakazakii* | 2 | — | — | — | — | — | — | — | — | — | — | — | 2 | 0.0 |
| *Enterobacter* *asburiae* | — | — | — | — | 4 | — | — | — | — | — | — | — | 4 | 0.0 |
| *Enterobacter* *cancerogenus* | 1 | — | — | — | — | — | — | — | — | — | — | — | 1 | 0.0 |
| *Enterobacter* *cloacae* | 15 | 14 | 20 | 7 | 9 | 13 | 20 | 19 | 21 | 26 | 31 | 11 | 206 | 2.3 |
| *Enterobacter* *hormaechei* | — | — | — | — | — | 1 | — | — | — | — | — | 1 | 2 | 0.0 |
| *Enterobacter* sp. | 3 | 2 | 4 | 3 | — | — | — | — | — | — | — | — | 12 | 0.1 |
| *Escherichia* *coli* | 242 | 243 | 187 | 248 | 227 | 313 | 373 | 281 | 432 | 378 | 486 | 305 | 3,715 | 41.5 |
| *Escherichia* *hermannii* | — | — | — | 1 | — | — | — | — | — | — | — | — | 1 | 0.0 |
| *Klebsiella* *aerogenes* | 13 | 9 | 8 | 8 | 5 | 4 | 7 | 10 | 8 | 3 | 11 | 11 | 97 | 1.1 |
| *Klebsiella* *oxytoca* | 2 | 5 | 5 | 4 | 3 | 5 | 9 | 11 | 14 | 4 | 27 | 4 | 93 | 1.0 |
| *Klebsiella* *pneumoniae* | 101 | 88 | 62 | 107 | 109 | 126 | 292 | 234 | 331 | 296 | 356 | 212 | 2,314 | 25.9 |
| *Klebsiella* *pneumoniae* subsp. *ozaenae* | — | 1 | 1 | — | 1 | 2 | — | 3 | — | 1 | 1 | 3 | 13 | 0.1 |
| *Klebsiella* sp. | — | 6 | 1 | 1 | — | — | — | — | — | — | 10 | — | 18 | 0.2 |
| *Morganella* *morganii* | 10 | 28 | 22 | 16 | 12 | 5 | 14 | 5 | 3 | 6 | 5 | 2 | 128 | 1.4 |
| *Pantoea* *agglomerans* | 1 | — | 1 | 3 | — | — | — | — | — | — | — | — | 5 | 0.1 |
| *Pluralibacter* *gergoviae* | — | — | — | — | 2 | — | — | — | — | — | — | 1 | 3 | 0.0 |
| *Proteus* *mirabilis* | 64 | 110 | 45 | 39 | 45 | 19 | 62 | 39 | 41 | 40 | 43 | 15 | 562 | 6.3 |
| *Proteus* *penneri* | 1 | 1 | — | 1 | — | — | 1 | — | — | 4 | 1 | — | 9 | 0.1 |
| *Proteus* sp. | 14 | 6 | — | — | — | — | — | — | — | 2 | — | — | 22 | 0.2 |
| *Proteus* *vulgaris* | 1 | 3 | — | — | — | — | — | 1 | — | — | — | 1 | 6 | 0.1 |
| *Providencia* *alcalifaciens* | — | — | — | — | — | — | 1 | — | — | — | — | — | 1 | 0.0 |
| *Providencia* *rettgeri* | — | — | — | — | — | — | — | — | 1 | — | 2 | — | 3 | 0.0 |
| *Providencia* *rustigianii* | — | — | — | 1 | — | — | — | — | — | 1 | 3 | — | 5 | 0.1 |
| *Providencia* sp. | 1 | 3 | — | 1 | — | 1 | — | — | — | — | — | — | 6 | 0.1 |
| *Providencia* *stuartii* | 35 | 40 | 23 | 8 | 5 | 10 | 11 | 11 | 8 | 7 | 1 | 5 | 164 | 1.8 |
| *Salmonella* *enterica* paratyphi A | — | — | — | — | 2 | — | — | — | — | — | — | — | 2 | 0.0 |
| *Salmonella* *enterica* subsp. *arizonae* | 1 | — | — | — | — | — | — | — | — | — | — | — | 1 | 0.0 |
| *Salmonella* sp. | — | — | — | — | — | — | — | — | — | — | — | 1 | 1 | 0.0 |
| *Serratia* *fonticola* | — | — | 1 | 3 | — | — | — | — | — | — | 2 | — | 6 | 0.1 |
| *Serratia* *liquefaciens* | — | 4 | 4 | 1 | 1 | — | — | — | 1 | — | 1 | — | 12 | 0.1 |
| *Serratia* *marcescens* | 5 | 5 | 5 | — | 2 | 5 | 5 | 13 | 9 | 9 | 12 | 14 | 84 | 0.9 |
| *Serratia* *odorifera* | 2 | 1 | — | — | — | — | — | — | 1 | — | 1 | — | 5 | 0.1 |
| *Serratia* *plymuthica* | — | 2 | 1 | — | — | — | — | — | — | — | — | — | 3 | 0.0 |
| *Serratia* sp. | — | 3 | 1 | — | — | — | — | — | — | — | — | — | 4 | 0.0 |
| *Shigella* *sonnei* | — | — | — | — | 2 | — | — | — | — | — | — | — | 2 | 0.0 |
| *Yersinia* *enterocolitica* | — | — | — | — | — | — | — | — | — | — | 1 | 1 | 2 | 0.0 |
| *Yersinia* *pseudotuberculosis* | — | 1 | — | — | — | — | — | — | — | — | — | — | 1 | 0.0 |
| Subtotal | 521 | 597 | 402 | 460 | 437 | 515 | 804 | 634 | 887 | 783 | 1,006 | 604 | 7,650 | 85.5 |
| Non-fermenting gram-negative bacilli | | | | | | | | | | | | | | |
| *Acinetobacter* *baumannii* | 51 | 31 | 20 | 20 | 29 | 24 | 45 | 33 | 29 | 43 | 63 | 18 | 406 | 4.5 |
| *Acinetobacter* *calcoaceticus* | — | 1 | — | — | — | — | — | — | — | — | — | — | 1 | 0.0 |
| *Acinetobacter* *haemolyticus* | — | 1 | — | — | — | — | 1 | — | — | 1 | — | — | 3 | 0.0 |
| *Acinetobacter* *lwoffii* | — | 2 | — | — | — | — | 3 | — | 3 | 1 | 3 | — | 12 | 0.1 |
| *Acinetobacter* sp. | — | — | — | — | — | 1 | — | — | — | — | 1 | — | 2 | 0.0 |
| *Alcaligenes* *faecalis* | 1 | — | — | — | — | — | — | — | — | — | — | — | 1 | 0.0 |
| *Burkholderia* *cepacia* | — | — | — | — | — | 1 | 1 | 3 | 1 | — | 3 | 1 | 10 | 0.1 |
| *Pseudomonas* *aeruginosa* | 84 | 49 | 68 | 62 | 45 | 54 | 83 | 48 | 77 | 79 | 107 | 34 | 790 | 8.8 |
| *Pseudomonas* *alcaligenes* | — | — | — | — | — | — | — | — | — | — | 1 | — | 1 | 0.0 |
| *Pseudomonas* *fluorescens* | 1 | — | 1 | 2 | 1 | — | 1 | 1 | — | 1 | 1 | — | 9 | 0.1 |
| *Pseudomonas* *luteola* | — | 1 | 1 | — | 1 | — | — | — | — | — | 1 | — | 4 | 0.0 |
| *Pseudomonas* sp. | — | 7 | 1 | 5 | 2 | 4 | 5 | — | 2 | 7 | 4 | 5 | 42 | 0.5 |
| *Stenotrophomonas* *maltophilia* | 1 | — | 2 | 2 | — | — | 2 | — | — | — | 1 | 1 | 9 | 0.1 |
| *Stutzerimonas* *stutzeri* | — | 1 | — | — | — | — | — | — | — | — | 2 | — | 3 | 0.0 |
| Subtotal | 138 | 93 | 93 | 91 | 78 | 84 | 141 | 85 | 112 | 132 | 187 | 59 | 1,293 | 14.5 |
| Total | 659 | 690 | 495 | 551 | 515 | 599 | 945 | 719 | 999 | 915 | 1,193 | 663 | 8,943 | 100.0 |
| ***Note.*** a—Trajectory-cohort urinary gram-negative bacilli cultures (from patients with at least two eligible cultures), 2013–2024. Em-dash (—) indicates zero isolates in that year.  b—2024 reflects a partial year; cultures were received only through 2024-09-16. | | | | | | | | | | | | | | |

| **Table S2.** Relative probability of same-species urinary gram-negative bacilli non-susceptibility persistence per doubling of time since the prior isolate | | | | | |
| --- | --- | --- | --- | --- | --- |
| Drug class and organism ᵃ | Non-susceptible follow-up / evaluable pairs, No. | Patients, No. | RR per doubling of time (95% CI) ᵇ | P value | Decline per doubling |
| Fluoroquinolone non-susceptibility | | | | | |
| *Escherichia* *coli* | 1506/1644 | 745 | 0.974 (0.968–0.980) | <0.001 | 2.6% relative reduction |
| *Klebsiella* *pneumoniae* | 714/794 | 379 | 0.978 (0.968–0.988) | <0.001 | 2.2% relative reduction |
| Extended-spectrum cephalosporin non-susceptibility | | | | | |
| *Escherichia* *coli* | 517/679 | 385 | 0.925 (0.909–0.941) | <0.001 | 7.5% relative reduction |
| *Klebsiella* *pneumoniae* | 507/565 | 310 | 0.964 (0.950–0.979) | <0.001 | 3.6% relative reduction |
| Carbapenem non-susceptibility | | | | | |
| *Escherichia* *coli* | 26/154 | 133 | Not estimable | — | Insufficient events |
| *Klebsiella* *pneumoniae* | 413/521 | 282 | 0.944 (0.924–0.964) | <0.001 | 5.6% relative reduction |
| Abbreviations: CI, confidence interval; GNB, gram-negative bacilli; RR, risk ratio. | | | | | |
| a—Main analyses are restricted to *Escherichia* *coli* and *Klebsiella* *pneumoniae,* the species meeting the support threshold; pooled pathogen-group and minority-species estimates are not shown as main inference (species-level support counts, Table S4).  b—Risk ratios were estimated using modified Poisson regression with patient-clustered robust standard errors. The RR is per doubling of time since the prior same-species isolate among prior-non-susceptible, class-evaluable same-species pairs; rows with insufficient persistent non-susceptibility events were not estimated. | | | | | |

| **Table S3.** Sensitivity and patient-balanced analyses of within-patient non-susceptibility persistence | | | | | |
| --- | --- | --- | --- | --- | --- |
| Sensitivity analysis, drug class, and organism ᵃ | Time since prior result, days ᵇ | | | | |
|  | 0–30 | 31–90 | 91–180 | 181–365 | >365 |
| A. Index isolate from inpatient setting | | | | | |
| Fluoroquinolone non-susceptibility | | | | | |
| *Escherichia* *coli* | 97.7 (311) | 95.8 (167) | 93.7 (63) | 77.2 (57) | 86.5 (74) |
| *Klebsiella* *pneumoniae* | 95.2 (314) | 91.8 (85) | 84.3 (51) | 79.2 (24) | 80.0 (25) |
| Extended-spectrum cephalosporin non-susceptibility | | | | | |
| *Escherichia* *coli* | 91.0 (210) | 83.1 (83) | 84.4 (32) | 54.2 (24) | 53.1 (32) |
| *Klebsiella* *pneumoniae* | 96.9 (262) | 90.1 (71) | 83.8 (37) | 69.2 (13) | 85.0 (20) |
| B. Index isolate from outpatient setting | | | | | |
| Fluoroquinolone non-susceptibility | | | | | |
| *Escherichia* *coli* | 100.0 (44) | 92.9 (42) | 84.2 (38) | 81.0 (21) | 90.9 (11) |
| *Klebsiella* *pneumoniae* | 78.9 (19) | 85.7 (14) | 87.5 (8) | 81.8 (11) | — |
| Extended-spectrum cephalosporin non-susceptibility | | | | | |
| *Escherichia* *coli* | 85.7 (14) | 55.6 (9) | 50.0 (8) | 40.0 (5) | 50.0 (2) |
| *Klebsiella* *pneumoniae* | 42.9 (7) | 100.0 (7) | 100.0 (4) | 80.0 (5) | 100.0 (2) |
| C. Index isolate from emergency or community setting | | | | | |
| Fluoroquinolone non-susceptibility | | | | | |
| *Escherichia* *coli* | 94.8 (232) | 95.9 (197) | 90.6 (127) | 86.8 (106) | 76.0 (154) |
| *Klebsiella* *pneumoniae* | 89.2 (93) | 91.5 (71) | 86.2 (29) | 81.5 (27) | 73.9 (23) |
| Extended-spectrum cephalosporin non-susceptibility | | | | | |
| *Escherichia* *coli* | 88.3 (77) | 68.5 (54) | 64.7 (34) | 59.1 (44) | 45.1 (51) |
| *Klebsiella* *pneumoniae* | 92.2 (64) | 87.9 (33) | 76.9 (13) | 75.0 (16) | 18.2 (11) |
| D. Restricted to ≥14-day inter-culture interval | | | | | |
| Fluoroquinolone non-susceptibility | | | | | |
| *Escherichia* *coli* | 94.8 (289) | 95.6 (406) | 90.4 (228) | 83.2 (184) | 79.9 (239) |
| *Klebsiella* *pneumoniae* | 90.0 (150) | 91.2 (170) | 85.2 (88) | 80.6 (62) | 77.1 (48) |
| Extended-spectrum cephalosporin non-susceptibility | | | | | |
| *Escherichia* *coli* | 86.3 (139) | 76.0 (146) | 71.6 (74) | 56.2 (73) | 48.2 (85) |
| *Klebsiella* *pneumoniae* | 93.4 (106) | 90.1 (111) | 83.3 (54) | 73.5 (34) | 63.6 (33) |
| E. Patient-balanced (equal patient weight) ᶜ | | | | | |
| Fluoroquinolone non-susceptibility | | | | | |
| *Escherichia* *coli* | 95.7 (93.3–97.4) | 93.9 (90.9–96.4) | 88.6 (83.3–92.7) | 80.9 (73.6–86.3) | 76.4 (69.9–81.7) |
|  | 96.8 | 95.6 | 90.4 | 83.2 | 79.9 |
| *Klebsiella* *pneumoniae* | 91.8 (88.1–94.4) | 87.7 (81.6–93.4) | 81.7 (71.4–90.5) | 80.2 (67.0–88.7) | 76.1 (63.6–87.5) |
|  | 93.2 | 91.2 | 85.2 | 80.6 | 77.1 |
| Extended-spectrum cephalosporin non-susceptibility | | | | | |
| *Escherichia* *coli* | 89.3 (84.5–93.0) | 71.7 (62.4–78.8) | 71.8 (57.7–80.3) | 51.0 (38.0–62.5) | 43.5 (31.8–53.9) |
|  | 90.0 | 76.0 | 71.6 | 56.2 | 48.2 |
| *Klebsiella* *pneumoniae* | 92.6 (88.4–95.6) | 88.8 (81.8–94.7) | 83.0 (68.1–89.4) | 72.7 (51.5–84.8) | 61.3 (38.7–74.2) |
|  | 94.9 | 90.1 | 83.3 | 73.5 | 63.6 |
| ***Note.*** a—Panels A-C stratify by collection setting of the prior non-susceptible isolate; Panel D restricts to consecutive same-species pairs separated by at least 14 days; Panel E gives equal weight to each patient. Analyses are restricted to *Escherichia* *coli* and *Klebsiella* *pneumoniae* and to fluoroquinolone and extended-spectrum cephalosporin non-susceptibility; carbapenem strata are not shown because support was sparse after stratification.  b—In Panels A-D, cells show the persistence percentage with the number of class-evaluable prior-non-susceptible same-species pairs in parentheses; an em dash indicates no evaluable pairs. These sensitivity analyses are descriptive.  c—In Panel E, the upper line shows the patient-balanced persistence percentage with a patient-clustered bias-corrected and accelerated bootstrap 95% confidence interval; the lower line shows the corresponding pair-level pooled percentage. Across cells, the median was one pair per patient (maximum, 2–13), so frequent re-culturers had limited influence. | | | | | |

| Table S4. Species-level support for organism-specific persistence estimates | | | | | | | | | | | | | | | | | | | |
| --- | --- | --- | --- | --- | --- | --- | --- | --- | --- | --- | --- | --- | --- | --- | --- | --- | --- | --- | --- |
| Species or group | Fluoroquinolones | | | | | Extended-spectrum cephalosporins | | | | | Carbapenems | | | | | Meets main-analysis display threshold ᵃ | | | Recommended role |
|  | 0–30 | 31–90 | 91–180 | 181–365 | >365 | 0–30 | 31–90 | 91–180 | 181–365 | >365 | 0–30 | 31–90 | 91–180 | 181–365 | >365 | FQ | ESC | Carb. |  |
| *Escherichia* *coli* | 587 | 406 | 228 | 184 | 239 | 301 | 146 | 74 | 73 | 85 | 71 | 34 | 20 | 17 | 12 | Yes | Yes | No | Main species; carbapenem non-susceptibility descriptive/not estimable |
| *Klebsiella* *pneumoniae* | 426 | 170 | 88 | 62 | 48 | 333 | 111 | 54 | 34 | 33 | 304 | 106 | 49 | 31 | 31 | Yes | Yes | Yes | Main (species-specific) |
| *Proteus* *mirabilis* | 118 | 54 | 23 | 12 | 17 | 85 | 26 | 21 | 6 | 4 | 6 | 3 | 3 | 0 | 3 | No | No | No | Support-count only (descriptive) |
| *Enterobacter* *cloacae* | 16 | 13 | 3 | 4 | 5 | 19 | 11 | 2 | 1 | 2 | 8 | 4 | 2 | 3 | 1 | No | No | No | Support-count only (descriptive) |
| *Providencia* *stuartii* | 20 | 11 | 3 | 4 | 0 | 17 | 15 | 2 | 1 | 1 | 6 | 1 | 0 | 0 | 0 | No | No | No | Support-count only (descriptive) |
| *Morganella* *morganii* | 9 | 9 | 5 | 2 | 2 | 10 | 8 | 2 | 2 | 2 | 1 | 1 | 0 | 0 | 0 | No | No | No | Support-count only (descriptive) |
| Other Enterobacterales | 27 | 20 | 5 | 3 | 4 | 18 | 15 | 5 | 2 | 1 | 16 | 6 | 1 | 1 | 3 | No | No | No | Support-count only (descriptive) |
| *Pseudomonas* *aeruginosa* | 87 | 33 | 17 | 13 | 11 | — | — | — | — | — | 74 | 28 | 13 | 11 | 9 | No | — | No | Support-count only (descriptive) |
| *Acinetobacter* *baumannii* | 65 | 11 | 7 | 6 | 6 | — | — | — | — | — | 55 | 5 | 6 | 5 | 6 | No | — | No | Support-count only (descriptive) |
| Other non-fermenting GNB | 1 | 0 | 0 | 1 | 0 | — | — | — | — | — | 0 | 0 | 0 | 0 | 0 | No | — | No | Support-count only (descriptive) |
| Abbreviations: Carb., carbapenems; ESC, extended-spectrum cephalosporins; FQ, fluoroquinolones; GNB, gram-negative bacilli. | | | | | | | | | | | | | | | | | | | |
| Cells are the numbers of class-evaluable prior-non-susceptible same-species pairs by species or group, drug class, and time since the prior isolate. An em dash indicates not applicable because extended-spectrum cephalosporin non-susceptibility is evaluable in Enterobacterales only.  The within-species pair cohort comprised 4332 same-species isolate pairs from 1745 patients; pair-cohort characteristics are summarized in the Results.  a—A species meets the main-analysis display threshold for a class if it has at least 30 evaluable pairs in at least 3 of 5 time intervals, including at least one interval beyond 180 days. Only *Escherichia* *coli* and *Klebsiella* *pneumoniae* meet the threshold; the remaining species are shown as support counts and were not analyzed as organism-specific estimates. Pathogen-group aggregates were not pooled because they would represent heterogeneous cohort mixtures rather than organism-specific persistence. | | | | | | | | | | | | | | | | | | | |

**Supplementary Table S5. CLSI M100 editions applicable by calendar year and summary of susceptibility-testing methods, 2013–2024.**

| **Study year** | **Applicable CLSI M100 edition** |
| --- | --- |
| 2013 | M100-S23 (23rd edition) |
| 2014 | M100-S24 (24th edition) |
| 2015 | M100-S25 (25th edition) |
| 2016 | M100-S26 (26th edition) |
| 2017 | M100, 27th edition (M100-Ed27) |
| 2018 | M100, 28th edition (M100-Ed28) |
| 2019 | M100, 29th edition (M100-Ed29) |
| 2020 | M100, 30th edition (M100-Ed30) |
| 2021 | M100, 31st edition (M100-Ed31) |
| 2022 | M100, 32nd edition (M100-Ed32) |
| 2023 | M100, 33rd edition (M100-Ed33) |
| 2024 | M100, 34th edition (M100-Ed34) |

Editions are listed by their nominal year of publication; in routine practice each edition was applied as adopted by the clinical microbiology laboratory during that year, and categorical interpretations reflect the edition in force at the time of testing. Because raw minimum inhibitory concentrations and zone diameters were not retained in the analytic dataset, isolates could not be retrospectively re-interpreted under a single harmonized breakpoint version.

**Susceptibility-testing methods (consistency note).** Organism identification and antimicrobial susceptibility testing were performed for clinical care throughout the study period on the VITEK 2 automated system (bioMérieux, Marcy-l’Étoile, France). The laboratory is accredited and performs routine internal quality control in accordance with CLSI-recommended procedures. Categorical susceptible/intermediate/resistant results were finalized at the time of testing under the year-appropriate CLSI M100 edition listed above and were collapsed to a binary non-susceptible (intermediate or resistant) scale for analysis. Specific VITEK 2 card/panel types, instrument software and Advanced Expert System (AES) versions, raw minimum inhibitory concentrations, disk-zone diameters, and isolate-level quality-control records were not retained in the analytic dataset; periodic card or software updates over the 12-year period cannot be excluded but are generally expected to have limited impact on categorical results.
