# Supplementary material for: Duration of same-species antimicrobial non-susceptibility persistence in urinary gram-negative bacilli: a 12-year within-patient cohort study
Source: Front Cell Infect Microbiol. 2026 Sep 11;16:1924619. doi: 10.3389/fcimb.2026.1924619 (PMC13612208; doi:10.3389/fcimb.2026.1924619)
Supplement: Supplementary file 2 [file Table2.docx]

# Supplementary Methods

## Cohort Construction and Pair Definition

Eligible cultures were urine cultures from patients aged at least 12 years that grew gram-negative bacilli from January 1, 2013, through September 16, 2024; 2012 was excluded as a partial first year, and external referrals were excluded. Patients with at least 2 eligible cultures formed the repeated-culture trajectory cohort for within-patient analysis.

Persistence was estimated using consecutive same-species pairs. Within each patient and species, isolates were ordered by collection date, and each isolate was paired with the next later isolate of the same species. Intervening cultures growing other species were skipped, because the target was same-species continuity rather than adjacent-culture recurrence. Time since the prior same-species isolate was grouped as 0-30, 31-90, 91-180, 181-365, and more than 365 days. Same-day same-species pairs were retained in the primary analysis; robustness analyses repeated estimation after restricting pairs to intervals of at least 14 days.

For each drug class, the persistence denominator was the number of same-species pairs in which the prior isolate was non-susceptible and the next isolate was class-evaluable. Persistence was the proportion of those class-evaluable follow-up isolates that remained non-susceptible. eTable 2 reports the prior-non-susceptible pair count, class-evaluable follow-up count, and patient count for each persistence cell.

## Non-susceptibility Class Specification

Drug-class phenotypes were non-mutually exclusive. A single isolate could be non-susceptible to more than one class and could contribute to more than one class-specific analysis when the relevant prior and follow-up isolates were evaluable for each class.

Fluoroquinolone non-susceptibility was defined using ciprofloxacin or levofloxacin. Extended-spectrum cephalosporin non-susceptibility was defined using cefotaxime, ceftriaxone, ceftazidime, or cefepime and was evaluated only for Enterobacterales. Carbapenem non-susceptibility was defined using organism-appropriate carbapenems. Difficult-to-treat non-susceptibility was not analyzed as a separate persistence class.

## Support Threshold and Not-Estimable Rule

Organism-specific persistence estimates were retained for main analytic display only when the species and class had at least 30 evaluable prior-non-susceptible same-species pairs in at least 3 of the 5 time intervals, including at least 1 interval beyond 180 days. Species not meeting this support threshold were not pooled for organism-specific inference, because aggregate pathogen-group estimates would combine biologically heterogeneous organisms.

This support criterion was investigator-defined and pragmatic rather than derived from a formal power calculation. The threshold of at least 30 evaluable pairs per cell aligns with the common rule-of-thumb for the normal approximation to a binomial proportion and was chosen to keep interval-specific proportion estimates reasonably stable and to mitigate small-sample instability; requiring coverage of at least 3 of the 5 intervals, including at least 1 interval beyond 180 days, ensured that temporal decay could be characterized across both short and long intervals rather than being anchored only to short-interval data.

For decay models, a class-by-species row was not estimated when there were fewer than 30 persistent non-susceptible follow-up outcomes or fewer than 30 nonpersistent follow-up outcomes. This rule was used to avoid unstable model estimates in sparse or near-boundary outcome distributions. Cell-specific proportions could still be summarized descriptively when model estimation was not supported.

## Estimation of Cell-Specific Persistence

The persistence point estimate was the pair-level pooled proportion within each organism, drug class, and time interval. The numerator was the number of class-evaluable follow-up isolates that remained non-susceptible to the same class. The denominator was the number of prior-non-susceptible same-species pairs whose next same-species isolate was class-evaluable.

Uncertainty for supported cells was estimated using patient-clustered bias-corrected and accelerated bootstrap confidence intervals with 10,000 replicates. Resampling occurred at the patient level over fixed pair rows so that all pair rows contributed by a sampled patient were retained together. Repeated sampling of the same patient within a replicate was handled with distinct re-indexing.

Wilson score intervals were used for sparse or boundary cells, defined as cells with an estimated proportion of at least 0.95 or at most 0.05, or fewer than 10 events in either outcome category.

## Modified-Poisson Decay Model

Temporal decay was estimated using modified-Poisson regression with a Poisson family, log link, and patient-clustered robust standard errors. The outcome was persistent non-susceptibility of the next same-species isolate to the same class among prior-non-susceptible, class-evaluable same-species pairs. The predictor was log_2_-transformed time since the prior same-species isolate.

Exponentiating the model coefficient gave the risk ratio for remaining non-susceptible per doubling of time since the prior isolate. A risk ratio was used rather than an odds ratio because persistence was common. Conditional logistic regression was not used.

## Applicability Analysis

The applicability analysis used adjacent-culture sequencing rather than same-species pair sequencing. For each class-specific index culture that was non-susceptible to the class and had a subsequent culture of any organism, the literal next culture was identified.

Two quantities were estimated: the proportion whose next culture grew the same species, and the proportion whose next culture grew the same species and was evaluable for the same class. Both proportions used index cultures with a subsequent culture as the denominator. Index cultures without a subsequent culture were excluded from these proportions and counted separately.

## Robustness Analyses and Software

Robustness analyses re-estimated persistence under three conditions: stratification by origin-of-index setting, restriction to same-species pairs separated by at least 14 days, and patient-balanced estimation giving equal weight to each patient within each organism, class, and time interval. Patient-balanced uncertainty used patient-clustered bias-corrected and accelerated bootstrap confidence intervals. Analyses were performed using StataNow/MP and Python.
